# Flexible Tactile Sensor Array for Slippage and Grooved Surface Recognition in Sliding Movement

**DOI:** 10.3390/mi10090579

**Published:** 2019-08-30

**Authors:** Yancheng Wang, Jianing Chen, Deqing Mei

**Affiliations:** 1 State Key Laboratory of Fluid Power and Mechatronic Systems, School of Mechanical Engineering, Zhejiang University, Hangzhou 310027, China; 2 Key Laboratory of Advanced Manufacturing Technology of Zhejiang Province, School of Mechanical Engineering, Zhejiang University, Hangzhou 310027, China

**Keywords:** finite element modeling, surface texture, grooved surface, tactile sensor array, wavelet transform, spectral analysis, inclined angle

## Abstract

Flexible tactile sensor with contact force sensing and surface texture recognition abilities is crucial for robotic dexterous grasping and manipulation in daily usage. Different from force sensing, surface texture discrimination is more challenging in the development of tactile sensors because of limited discriminative information. This paper presents a novel method using the finite element modeling (FEM) and phase delay algorithm to investigate the flexible tactile sensor array for slippage and grooved surfaces discrimination when sliding over an object. For FEM modeling, a 3 × 3 tactile sensor array with a multi-layer structure is utilized. For sensor array sliding over a plate surface, the initial slippage occurrence can be identified by sudden changes in normal forces based on wavelet transform analysis. For the sensor array sliding over pre-defined grooved surfaces, an algorithm based on phase delay between different sensing units is established and then utilized to discriminate between periodic roughness and the inclined angle of the grooved surfaces. Results show that the proposed tactile sensor array and surface texture recognition method is anticipated to be useful in applications involving human-robotic interactions.

## 1. Introduction

Flexible tactile sensors have been widely utilized in robotics, prosthetic hands, and medical surgery [1,2]. For grasping and manipulation tasks, the robotic hand with integrated tactile sensors can perceive tactile information between the hand, fingers, and grasped objects. This tactile information plays an important role and can be used for robotic feedback control [3,4]. For daily grasping in robotic and prosthetic hands, if the applied grasping force is too low, objects may slip through the hand, while fragile objects may be damaged when the applied force is too large. Furthermore, the roughness, texture, material hardness, and contour of the objects also affect the requisite grasping force [5]. Therefore, robotic dexterous manipulation generally requires integrated tactile sensors on the robotic hand with force sensing as well as object texture and contour shape recognition abilities.

The tactile sensor array is usually designed with several sensing units arranged in a row/column configuration and can be used to measure distributed contact forces [6,7]. In the past decade, developments in the tactile sensor array have attracted many researchers, and several types of tactile sensor array have been proposed [8,9,10]. Recently, we utilized conductive rubber as the sensing material to develop a flexible tactile sensor array with 3 × 3 sensing units which can be worn on the finger of a robotic hand and can measure three-axis contact forces during grasping applications [11,12]. The contact behavior of the sensor array with objects affects the contact force sensing performance of the tactile sensor array we developed. Analytical modeling was conducted to study the sensing performance and mechanical behavior in many researches. The basic structure of the tactile sensor is usually first simplified into a combination of cylinders, cuboids and other basic geometries. Then a lumped parameter model can be developed to analyze the mechanical properties of the tactile sensor. Zhang et al. [13] utilized the Stribeck friction model to study the mechanical behavior between a rigid gripper and the gripped object during the initial slippage phase. They found that the induced normal force changes suddenly when slipping occurs due to change in the static/dynamic friction coefficient. Ho et al. [14,15] simplified the fingertip into a bundle of beam to calculate localized displacement for slip detection. 

For a tactile sensor array with a more complex structural design, numerical modeling will be an effective approach to study the performance and contact behavior of the tactile sensors. Dao et al. [16] presented a numerical model in Marc Mentat software that analyzed the normal stress distribution of the sensing units when an external force is applied and identified the optimal location for the arrangement of piezoresistors. Youssefian et al. [17] developed a finite element modeling (FEM) of the tactile sensor by adopting nonlinear elastic material properties to study the induced stress and strain when a normal force is applied to the outer surface of the tactile sensors. In these proposed numerical models, the structure of the tactile sensors needs to be simplified into beam and plate structures for fast calculation convergence. Therefore, this will inevitably affect the accuracy of the mechanical behaviors of the tactile sensors for external force sensing, like the filtering effects of the sensor’s top cover material is neglected [18]. Thus, to analyze the sensing performance and contact behavior, an accurate 3D FEM model of the tactile sensor array needs to be developed, and this is a goal of this research. 

As mentioned earlier, the surface roughness, hardness, texture and contour shape of the object affect the sensing performance of the tactile sensor array. For surface texture recognition, two approaches have been validated to have the ability to extract the object’s features. (1) Using a tactile sensor array with high-density sensing units to measure the contact forces when it touches the object’s surface. Then the measured force values are plotted into a gray scale figure, which can be used to discriminate between the contour shapes of the objects using an image processing algorithm [19,20]. (2) Using a spectral analysis algorithm to analyze the measured forces when the tactile sensor slides along the surface of the objects. Oddo et al. [21] utilized a 2 × 2 tactile sensor array to measure the normal forces when sliding over the patterned surfaces, and fast Fourier transformation (FFT) was used to discriminate between the surface roughness and periodic information from the grooved surfaces. Further, they developed an approach using a machine learning algorithm (k-NN classifier) and wavelet transform to classify the surface texture [22]. In 2012, Fishel et al. [23] utilized a biologically inspired tactile sensor (BioTac) to measure tactile vibrations and reaction forces when exploring surfaces with different textures. The Bayesian exploration algorithm was then used to analyze the force data obtained, and 117 types of textures were successfully identified. This algorithm requires plenty of input data for the improvement of accuracy, and this limits the application of the BioTac sensor for surface texture recognition. Therefore, based on the obtained reaction forces of the tactile sensor array, an effective surface texture recognition method still needs to be developed. 

Therefore, the proposed tactile sensor for surface texture recognition still needs to be investigated, the present surface recognition method can still not be used for practical usage. To fill this research gap, we developed an accurate 3D FEM model of the tactile sensor array to study the sensing performance and contact behavior of the sensor when contacted with objects. Based on the measured contact forces of the tactile sensor array, a novel approach based on phase delay algorithm for grooved surface recognition is developed and verified by both FEM modeling and experimental validation. The main content of this paper is divided as follow: Section 2 presents the structure and working principles of the tactile sensor array on which 3D FEM modeling was conducted. Section 3 describes the experimental setup and procedures. Two sets of experiments were conducted: slippage detection and surface texture recognition when the sensor array slides over the object’s surface. The FEM simulation, experimental results, and discussion are presented in Section 4.

## 2. Design of Tactile Sensor Array and FEM Modeling

### 2.1. Flexible Tactile Sensor Array

**Figure 1:**
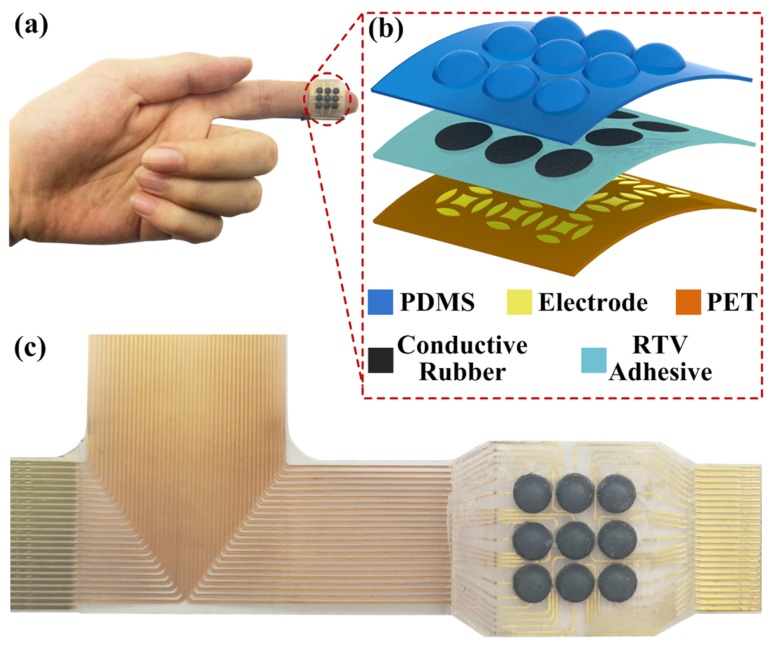
(**a**,**b**) Structure of the flexible tactile sensor array; (**c**) Fabricated tactile sensor array.

Of the tactile sensing principles, piezoresistive sensing is selected because of its relatively simple structural design and good anti-noise performance. Highly sensitive INASTAMOR pressure conductive rubber (from Inaba Rubber Co. Ltd., Osaka, Japan) is utilized as the sensitive material and cut into small pieces of round-shaped chips with a diameter of 3.0 mm. The structural design of the 3 × 3 flexible tactile sensor array is illustrated in Figure 1a,b. This sensor array mainly consists of three layers: top polydimethylsiloxane (PDMS) bump, a middle room temperature vulcanizable (RTV) adhesive layer with conductive rubber chips, and bottom electrodes on a thin film of polyethylene terephthalate (PET). The thicknesses of these three layers are 0.8, 0.5 and 0.1 mm, respectively. The distance between adjacent units is about 3.5 mm, and thus the overall dimensions of the tactile sensor array are 20 mm × 16 mm × 1.4 mm. A detailed structural design of the flexible tactile sensor array can be found in one of the references [12].

The patterned electrodes underneath the rubber chip have four side electrodes and one central common electrode, which generates four resistors (*R*_1_, *R*_2_, *R*_3_ and *R*_4_) and divides the sensing unit into five areas, as shown in Figure 1b. Thus, these four resistors can measure the changes in resistance for external three-axis force sensing. Typically, as the tactile sensor array is worn on the finger of a hand for grasping and touching objects, the external force will be exerted over the sensor array, and the induced deformation of the PDMS bump and conductive rubber chips will change the resistances of these four resistors.

### 2.2. FEM Modeling

**Figure 2:**
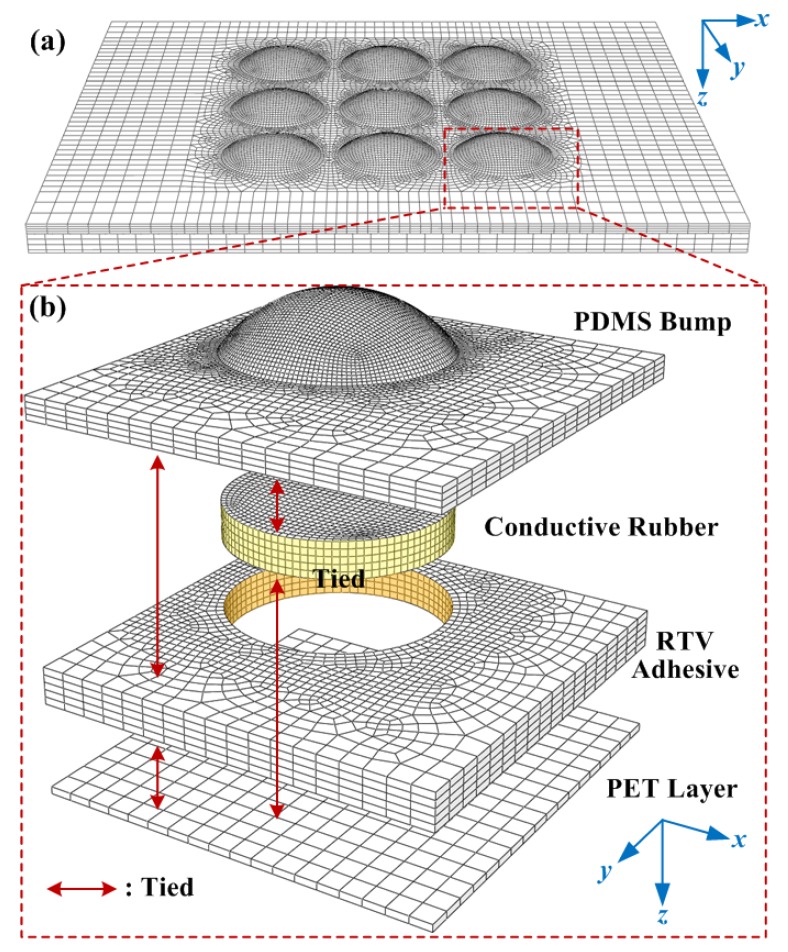
(**a**) 3D finite element modeling (FEM) model of flexible tactile sensor array; (**b**) Close-up view of sensing unit.

For FEM modeling, the finite element mesh of the 3D tactile sensor array model with dimensions of 20 mm × 16 mm × 1.4 mm is shown in Figure 2a. The accurate 3D geometry of the bump, rubber chip, and substrate film layers were converted to the FEM mesh using ABAQUS (v6.14, Dassault Systèmes Simulia Corp., Providence, RI, USA). Both “structured” and “sweep” algorithms for element mesh were utilized. The bump layer, rubber chip and surrounding RTV adhesive, and the PET film were connected using the “tie” function to lock the nodes onto the surfaces. To ensure perfectly tied surfaces, the mesh (node positions) on the mating surface must be consistent. For mesh convergence, the region of the bump and rubber chip and other contact regions were finely meshed, as shown in Figure 2b. Altogether, the tactile sensor array was meshed using 78,885 eight-node hexahedron elements. The element numbers in each layer are as follows: bump (53,236 elements), conductive rubber chip (10,368 elements), RTV adhesive (14,001 elements) and bottom PET film (1280 elements). For boundary conditions, the PDMS bump, conductive rubber chip, RTV adhesive, and PET film layers were merged together. The underside of the PET layer was confined by the displacement boundary condition.

**Figure 3:**
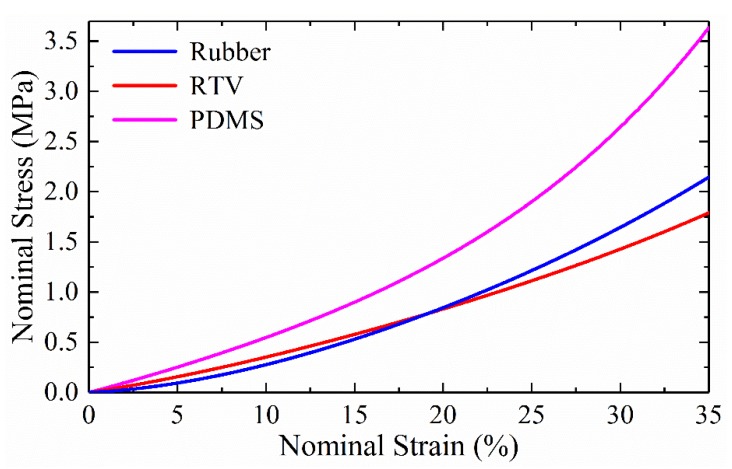
Measured nominal stress versus strain curves of rubber, room temperature vulcanizable (RTV) and polydimethylsiloxane (PDMS) materials.

The mechanical properties of the PET film were adopted from a previous study while Young’s modulus and Poisson ratio are about 3000 MPa and 0.47 [24]. For the conductive rubber, PDMS and RTV adhesive, uniaxial compression tests were conducted according to American Society for Testing and Materials (ASTM) standards [25]. The measured nominal stress versus nominal strain curves for the rubber, PDMS and RTV adhesive materials are shown in Figure 3.

All three stress-strain curves have nonlinear elastic behaviors, especially under large strains. The hyper-elastic Yeoh model [26] was used in the ABAQUS software to represent the nonlinear properties of the conductive rubber, PDMS and RTV adhesive materials. For these incompressible materials, the strain energy density function can be expressed as

(1) $$W(I_{1})=C_{1}(I_{1}-3)+C_{2}{\left(I_{1}-3\right)}^{2}+C_{3}{\left(I_{1}-3\right)}^{3}$$

where *I*_1_ stands for the first invariant of the Green deformation tensor and *C_i_* is the material parameter. Under the circumstance of uniaxial compression, Equation (1) can be transformed into the form that describes the relation between the stress *σ* and strain *ε* as

(2) $$\begin{array}{l} \sigma =2C_{1}(\lambda -\lambda^{-2})+4C_{2}(\lambda^{3}-3\lambda +1+3\lambda^{-2}-2\lambda^{-3})\\ +6C_{3}(\lambda^{5}-6\lambda^{3}+3\lambda^{2}+9\lambda -6-9\lambda^{-2}+12\lambda^{-3}-4\lambda^{-4}) \end{array}$$

where *λ* is the elongation and equals to 1 + *ε*.

By using the least squares fitting, three parameters (*C*_1_, *C*_2_, and *C*_3_) in the Yeoh model for rubber, RTV adhesive, and PDMS are obtained, and these are listed in Table 1.

## 3. Experimental Setup and Procedure

### 3.1. Experimental Setup

**Figure 4:**
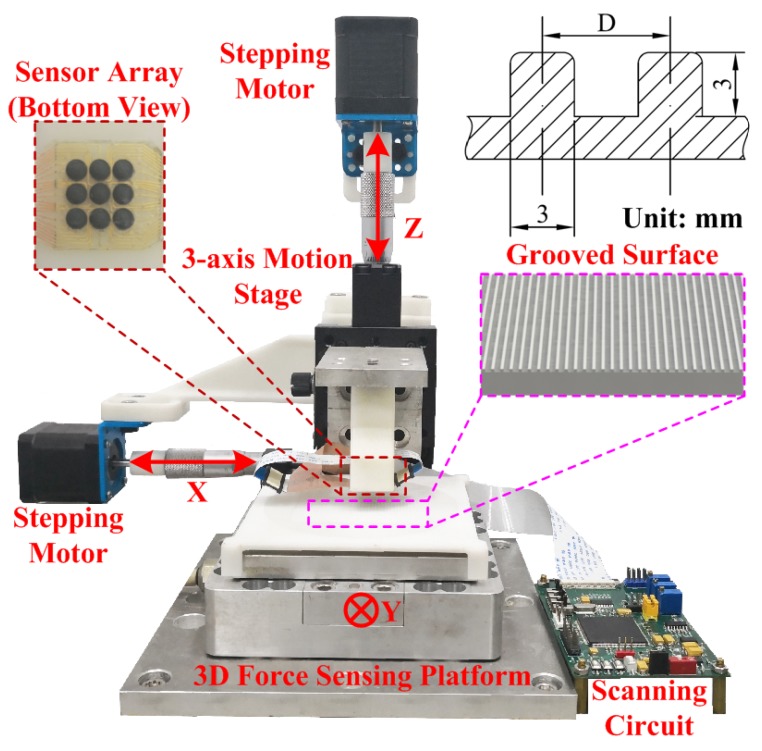
Experimental setup for the validation tests.

The entire experimental setup is shown in Figure 4. It mainly consists of an *xyz* linear motion stage and a three-axis commercialized force sensor. The developed flexible tactile sensor array was attached to a plastic loading bar, which was mounted to a *z*-axis motion stage. A scanning circuit based on digital signal processing (TMS320F2812, Texas Instruments Inc, Dallas, TX, USA) was designed and used for the distributed normal and shear forces measurement [12]. During the experiments, two types of surfaces (flat surface and grooved surface) made using stereolithography (SLA) technique were utilized. The flat surface was used for slippage detection when the loading bar and tactile sensor array slid on the surface. The grooved surfaces with different patterns were used for surface texture recognition.

### 3.2. Experimental Procedure

During the experiments, the motion speed of the linear stage was controlled by the stepping motors. To reduce the inertia effects, the sliding movement of the loading bar and tactile sensor array should be lower than 1.0 mm/s. For experimental validation, two sets of experiments were conducted:

***Slippage detection.*** First, the loading bar with a tactile sensor array compressed the flat surface for 8 s, and the induced compression force was increased up to 20 N. This force was a little large than that of FEM simulation, because this force is sufficient to overcome the effect of the death zone, and make the output voltage of each sensing unit clear enough (over 0.5 V) for further analyses. Secondly, this is followed by a holding stage that lasted for 15 s. Thirdly, the loading bar and tactile sensor array slid along the plate surface for about 25 s at a constant speed of 0.25 mm/s.

***Surface texture recognition.*** Three grooved surfaces with different spatial periods of 0.9, 1.2, and 1.5 mm were utilized. The inclined angle (*α*), defined as the angle between the grooves and the *y*-axis, was set as 0–60° with an increment of 15°. The tactile sensor array first compressed the grooved surface for about 3 s, and then held for 10 s. The induced compression force was also kept as 20 N. Then, the loading bar and tactile sensor array slid along the grooved surfaces for about 40 s.

The sampling rate of the scanning circuit for the generated forces sensing was set as 0.1 kHz. For each experimental set, three repeated tests were carried out repeatability.

## 4. Results and Discussion

### 4.1. Slippage Detection in Sliding Movement

By using the developed FEM model, the sliding movement of the tactile sensor array over the flat surface was analyzed. During the simulation, the sliding movement is divided into two steps. Step I—compressing and holding: the tactile sensor array was vertically compressed against the flat surface until the total reaction force reaches up to about 15 N. This force was a little lower than that of experimental tests with the aim to improve the convergence property and the convergence effectiveness of sliding movement during FEM simulation. As for each sensing unit, the force difference between experimental and FEM simulation can be further decreased to about 0.56 N. Then this step was held for 1 s to maintain the normal force of 15 N. Step II—sliding: the sensor array was moved sliding along the *x*-axis direction at a speed of 1.0 mm/s. Because our developed tactile sensor array has extremely low weight less than 10 g, the moving speed lower than 1.0 mm/s can be considered as the quasi-static state movement. So, the moving speed lower than 1.0 mm/s has little effect on the FEM simulation predicted forces and will greatly reduce the simulation time.

**Figure 5:**
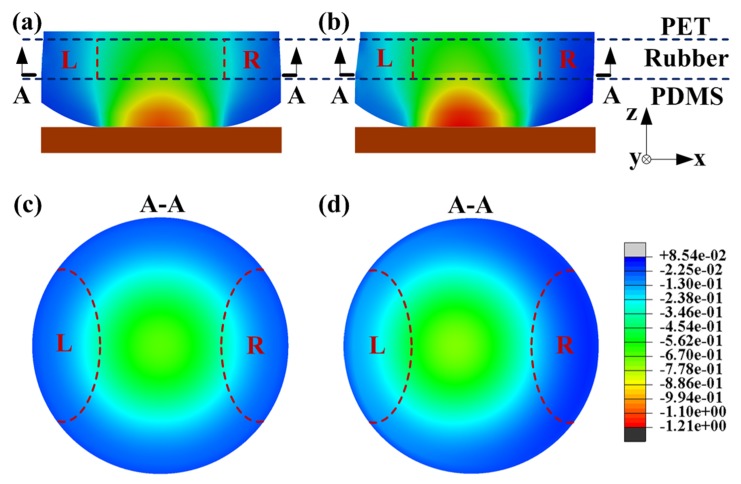
The stress distribution in the cross-section view of the sensing unit at the end of (**a**) compress and (**b**) sliding. A-A cross-section at the end of (**c**) compress and (**d**) sliding.

FEM simulation results for each sensing unit at the end of the compression and sliding stages are shown in Figure 5. In Figure 5a,c, the generated normal stress along the *z*-axis is symmetrically distributed at the cross-section view of the sensing unit during the compression stage. During the sliding stage, the compression stress in the left region (marked as *L*) is generally increased and becomes greater than that in the right region (marked as *R*), as shown in Figure 5b,d. This phenomenon has been confirmed by other studies [11,12] and can be attributed to the torque caused by the friction between the flat plate and the PDMS bump. The shear deformation of the sensing unit also occurred during sliding movement. This can be explained as the localized displacement occurred at the contact region of the PDMS bump [14,15]. The lower boundary of the hemisphere-shaped bump cannot immediately follow the movement of the upper part of the tactile sensor due to the friction in the contact region. Thus, gross slippage will be generated as the distance between the contact region and the PET substrate is stretched long enough to overcome the effects of friction.

**Figure 6:**
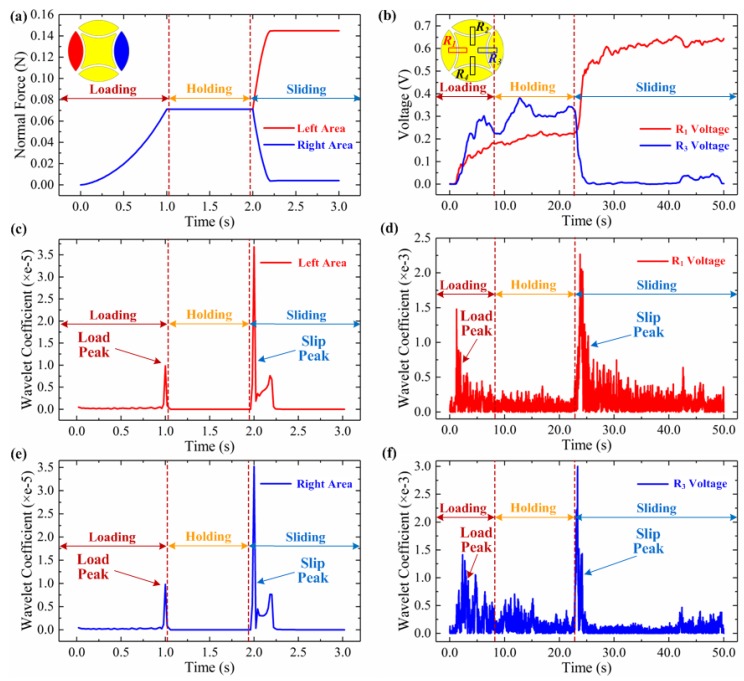
(**a**) Simulated normal force extracted from the left and right area of the patterned electrodes in the sensing unit (**b**) Measured voltages of *R*_1_ and *R*_3_ when sliding along a flat surface, DSWT analysis of the simulated normal force in left area (**c**) and right area (**e**) DSWT analysis of the measured voltages in *R*_1_ (**d**) and *R*_3_ (**f**).

The normal force generated at the left and right areas of the sensing unit are extracted based on the simulation results, as shown in Figure 6a. In Figure 6a, the sliding direction of the tactile sensor array along the flat surface can be observed as from the red area to the blue area. The normal forces obtained at both the left and right area are gradually increased from zero to 0.07 N at the loading stage. During the sliding stage, the normal force at the left area is increased significantly, while the generated normal force in the right area is decreased. Though the force’s amplitude was generally small, the relative change rate almost reaches 100%. Therefore, the sliding direction can be identified based on the obtained normal force curves in the left and right areas.

To validate the FEM prediction, experimental tests are conducted. Figure 6b shows the measured voltages of the tactile sensor array in *R*_1_ and *R*_3_ resistors when the sensor array is compressing and sliding on a flat surface. The experimental setup and procedure adopted are presented in the preceding Section 3. Generally, the measured voltages of *R*_1_ and *R*_3_ have greater variations while having almost the same trends as that of the simulated normal force, as shown in Figure 6a. This is because the sensitivity of the utilized conductive rubber material in tactile sensor is over 500 kΩ /N when the applied force is lower than 0.7 N [12]. At the sliding stage, the measured voltage of *R*_1_ is also increased, and the voltage of *R*_3_ is decreased. Therefore, we can clearly distinguish the sliding occurrence and direction from either the FEM simulated normal forces or the measured voltages at the side electrode area and resistors.

Initial slippage detection is proven to be important for robotic hand grasping. Wavelet transform has been utilized to analyze the measured forces or voltages of the tactile sensors and demonstrates the ability to identify the change of the derivation for initial slippage discrimination [28]. Here, we also utilized the wavelet transform to analyze the simulated normal forces and measured voltages in Figure 6a,b. Figure 6c–f shows the discrete sequence wavelet transform (DSWT) results of the simulated normal forces and measured voltages of *R*_1_ and *R*_3_, respectively. We picked Coiflet as the mother wavelet function and set its length equal to 6. Two peaks can be observed at the transition moments from the loading to holding and from the holding to sliding stages, as shown in Figure 6c–f. The variations of the wavelet coefficient at initial sliding are much greater than that after loading. Thus, by setting a reasonable threshold value for the wavelet coefficient, the initial slippage can be distinguished as the tactile sensor array contacts and slides along object surfaces. More details of this method for slippage detection can be found in our previous study [11].

### 4.2. Surface Texture Recognition

#### 4.2.1. Phase Delay Algorithm for Surface Texture Recognition

**Figure 7:**
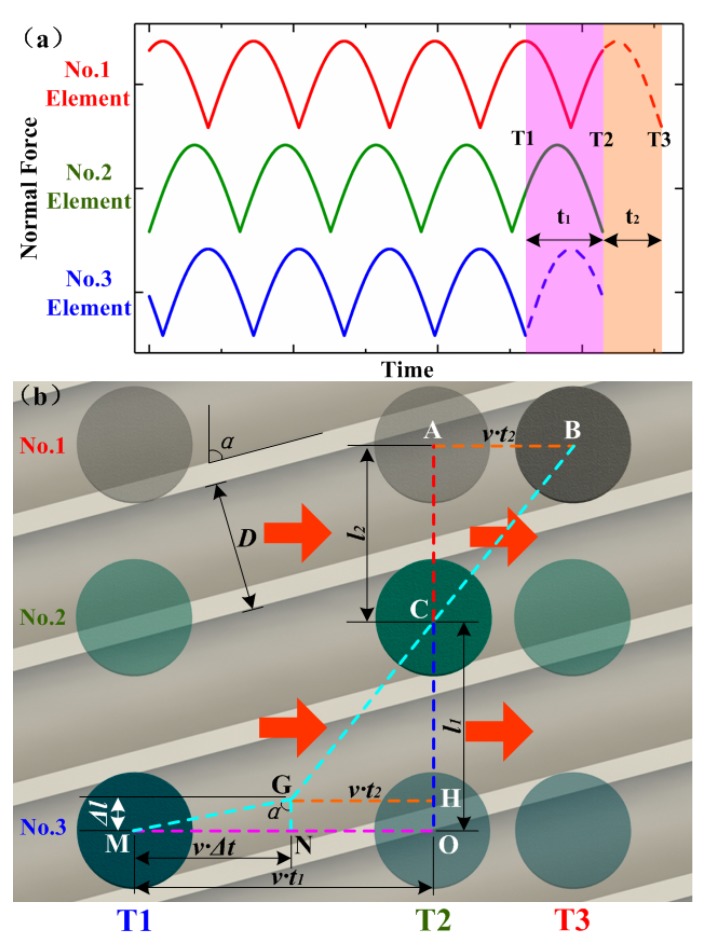
(**a**) Force-time curves of No. 1–3 units, (**b**) schematic view for the calculation of inclined angle α.

Spectrum analysis of the tactile sensor’s output voltages can be used to determine the spatial periodical information of the utilized grooved surfaces. The spatial period value (*D*), defined as the distance between two adjacent ridges (as shown in Figure 7b), can be calculated as:

(3) D=v/MAF

where *v* is the sliding speed, and MAF stands for the frequency with the maximum amplitude.

In practical application, the grooved texture in the plate surface usually has an inclined angle of *α*. Applying FFT to the obtained data may get us a fake spatial period value equal to *D*/cos(α). Therefore, the inclined angle (*α*) also needs to be determined. For this purpose, we changed the distance between the sensing units in each row and column to create different phase delays, as shown in Figure 7b. We assumed that the force-time curves of one column of three sensing units are as shown in Figure 7a. The horizontal movement ends at *T*_2_, when No. 2 unit is at the center of the groove, which causes the force-time curve to end at a minimal value. At *T*_1_, the No. 3 unit will also be in a similar position where it sustains the lowest pressure. If the sliding movement continues, the force of No. 1 unit will drop to the same value at T_3_. In this condition, the movement path distances (AB and MO) can be calculated as *vt*_1_ and *vt*_2_, respectively, where *t*_1_ and *t*_2_ are the gaps between each moment, as indicated in Figure 7a. If the distance AC (*l*_1_) or CO (*l*_2_) is longer than that of *D*/sin(*α*), the inclined angle (*α*) can be calculated using the anti-trigonometric function and can be expressed as

(4) $$\alpha =\arctan(\frac{v\cdot t_{1}}{l_{1}})$$

The schematic diagram to calculate α is shown in Figure 7b. △CGH is first created as the same as △CBA, where point H is located on line CO. Then, we connect point G and point M and create a right triangle △GNM, where line GN is perpendicular to line MN. The angle ∠MGN is the same as that of inclined angle *α*. The length of GN and MN can be calculated as (*l*_1_*−l*_2_) and *v·*(*t*_1_*−t*_2_), respectively. Thus, the inclined angle *α* can be calculated as

(5) $$\alpha =\arctan[\frac{v\cdot (t_{1}-t_{2})}{l_{1}-l_{2}}]$$

Also, the spatial period value (*D*) can be calculated as

(6) D=v⋅cos(α)/MAF

**Figure 8:**
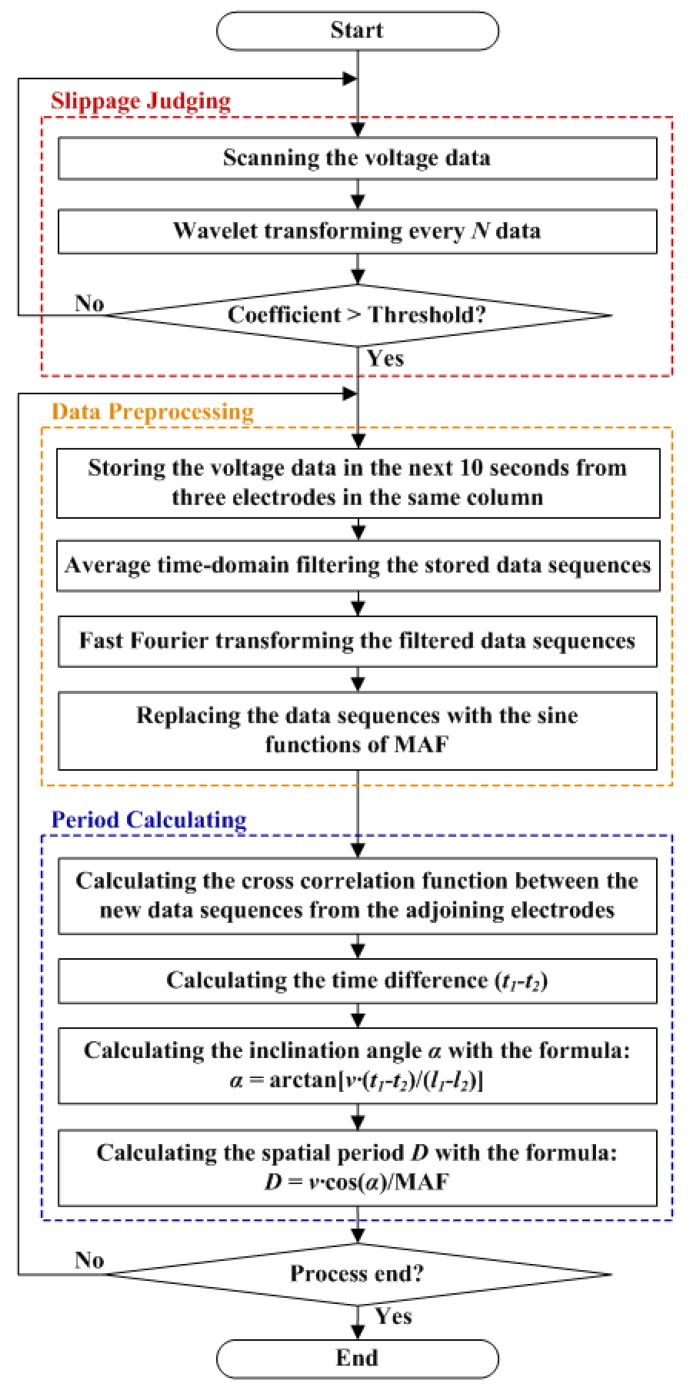
Flow chart and procedure to calculate the inclined angle and spatial period value for the grooved surfaces.

The procedure and flow chart of the phase delay algorithm for the grooved surface texture recognition is shown in Figure 8. The whole procedure can be mainly divided into three modules. In Slippage Judging module, the threshold based on wavelet coefficient is used. As the coefficient value is larger than 0.002, the program will jump out of the first loop and enter the Data Preprocessing module. The scanning circuit will sample the voltage data from three electrodes in the same column for 10 s. Using the low-pass filtering, the time gap (*t*_1_ and *t*_2_) is calculated based on the cross-correlation function analysis in this step. As for simulation, the force curves usually have an approximate sinusoidal shape. As for real tests, the measured voltage signals will be affected by external noises and vibrations, making the voltage curves not as good as that of the simulation results. Thus, we reconstructed the sine function of the characteristic frequency in the time domain and input these new curves into the cross-correlation function as shown in Figure 8. This step can be regarded as “band-pass filtering”, which can improve the accuracy of the final results. After getting the reconstructed sine function, the program enters the Period Calculating module. By using Equations (5) and (6), the inclined angle (*α*) and spatial period value (*D*) can be calculated. Both maximum and minimum points of the cross-correlation curve will be taken as two different inputs. Therefore, we can obtain two spatial period results in the end of this module. If the difference between these two values is greater than 0.01 mm, the program will jump back and sample another set of data for a new round of calculation. Otherwise, the mean value will be output.

#### 4.2.2. Spatial Period Discrimination

**Figure 9:**
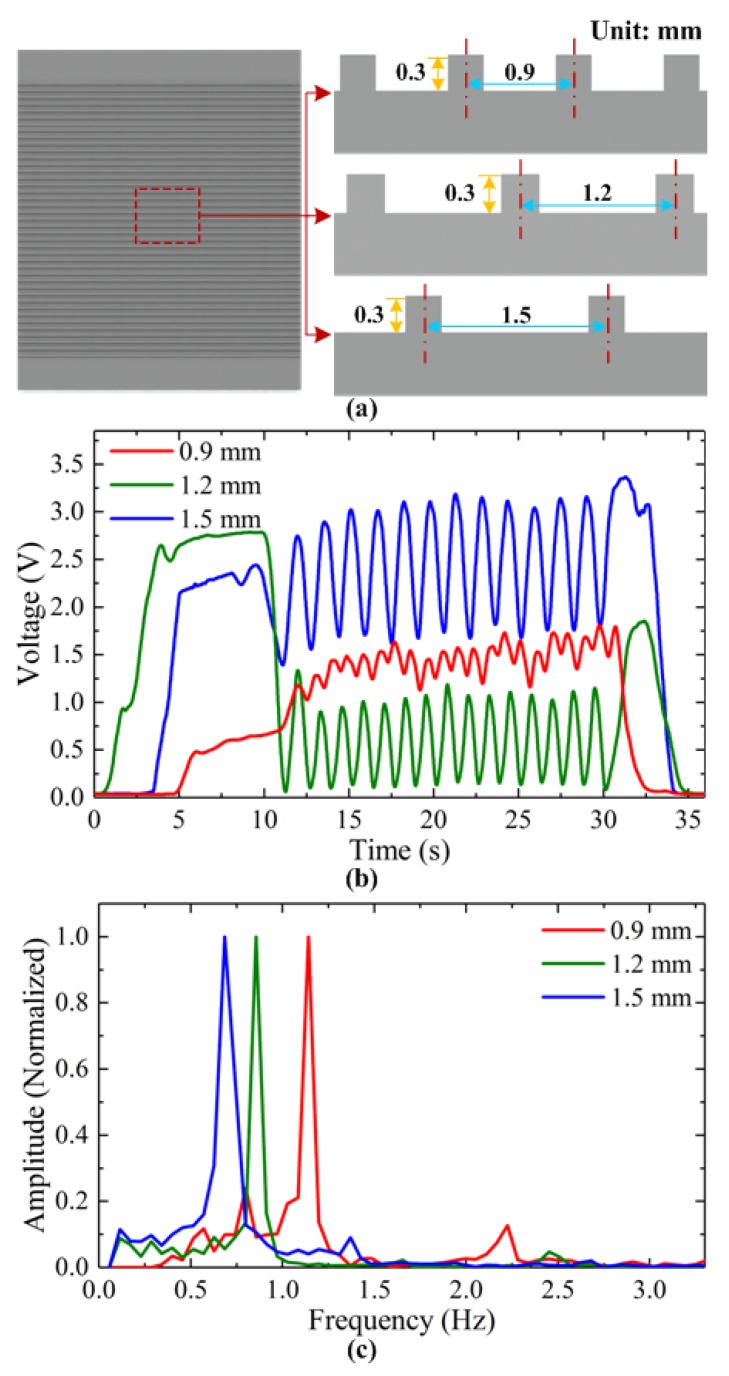
(**a**) Three grooved surfaces with different spatial periods of 0.9, 1.2 and 1.5 mm; (**b**) Measured voltage; (**c**) Spectrum analysis for surface texture recognition.

To verify the ability of the developed method for surface texture recognition, both simulation, and experimental tests were performed when the tactile sensor array slid along the grooved surfaces. For grooved surface recognition, the compression force was set as 15 N and 20 N for FEM simulation and experimental tests, respectively. The applied force during experimental tests is a little larger. The reasons are the measured output voltage of our tactile sensor array usually contains some noises from the scanning circuit and environment. If the applied force is too small, it will affect the accuracy of tactile sensor for surface texture recognition. Even the misjudging of the frequency characteristics may be occurred, and leads to false result. For the utilized grooved surfaces, as shown in Figure 9a, the inclined angle of the grooves on the plate is set as zero and spatial period as 0.9, 1.2 and 1.5 mm, respectively. As the sensor array slid along the surface, the measured voltages of one sensing unit for these grooved surfaces are shown in Figure 9b. We can see that the variation of voltage signals increases with the increase in the spatial period. For example, the grooved surface with 1.5 mm spatial period has the highest variation in the voltage curve. It is because narrower groove makes the compressed bumps of sensor less released.

Using the calculation procedure in Figure 8 (simplified, as there is no inclined angle), spectrum analysis is conducted, as shown in Figure 9c. For comparison, the MAF and spatial periods of the grooved surfaces in simulation and experimental tests are calculated and listed in Table 2. We can see that the deviation of the calculated MAF and the spatial period from the real values are generally low as the errors are usually smaller than 6.7%. Thus, we can conclude that plate surfaces with different grooved textures can be recognized successfully using the method developed and the proposed tactile sensor array.

#### 4.2.3. Inclined Angle Calculation in Grooved Surfaces

To validate the developed method for the calculation of grooved surface with an inclined angle, we set *l*_1_ and *l*_2_ as 3.5 mm and 3.8 mm, respectively. For simulation and experimental tests, the grooved surface’s spatial period is set as 1.2 mm and the inclined angle of the grooved patterns as 0°, 15°, 30°, 45° and 60°, respectively.

According to the calculation procedure and Equations (5) and (6), the inclined angle and spatial period for simulation and validation tests are calculated as shown in Table 2. We can see that both the calculated inclined angle and spatial period values for the simulation and experiments generally match well with the pre-determined results. For angle calculation, the greatest errors (23.3% for the simulation and 7.4% for the experiment) occur when the inclined angle equals 15°. It is because the slope of the inversed tangent function is extremely steep when the inclined angle is lower than 20°. Thus, even a small difference in the inputs would affect the accuracy of the calculated results. For the angle in the range of 30° to 60°, the relative errors are greatly reduced and less than 5.0%, as shown in Table 2. For the spatial period calculation, as it is also influenced by the sampling time, the error’s variation shows a different trend compared with the previous one. Still, the biggest error is less than 9.2% for both simulation and experiment tests. Therefore, a plate with inclined arranged grooves on the surface can also be recognized using the developed method.

**Figure 10:**
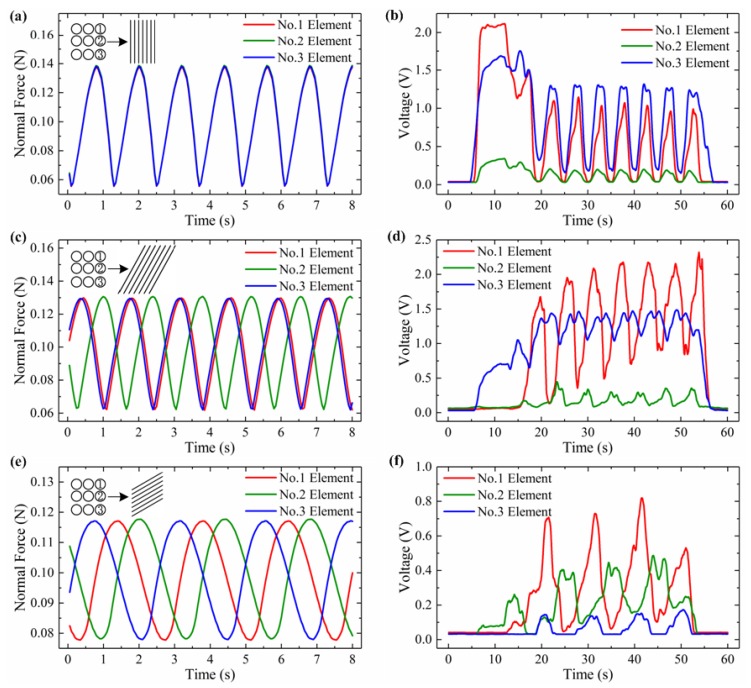
Simulated normal force curves when the sensor array is sliding along the grooved surface with an inclined angle α equal to (**a**) 0°, (**c**) 30° and (**e**) 60°. Measured voltages from the validation test for inclined angle calculation when α equals (**b**) 0°, (**d**) 30°, (**f**) 60°.

Typically, the simulated force curves for the inclined angle of 0°, 30° and 60° are shown in Figure 10a,c,e. The peak number in the force curve is decreased from 7 to 6 when the inclined angle increased from 0° to 30°. The peak number dramatically drops to 3 when the angle is raised up to 60°. This is due to the inclined grooves will enlarge the horizontal gap distance between the adjacent grooves. Under a uniform sliding motion, the larger angle will increase the time period, and in turn leads to less peaks, as show in Figure 10a,c,e. These three figures also show the trends that the overlapped force curves are gradually apart from each other. The green one is much closer to the red one than that of blue one. This phenomenon verifies that the existence of phase delay which caused by the inclined arranged grooves. Longer distance between elements 2 and 3 makes the phase delay more obviously. As in Figure 10e, the variation of the force curves drops from 0.08 N to 0.04 N with the increase of inclined angle. The curves stand for the total normal force applied on a fusiform area as shown in Figure 5. The inclined grooves arrangement will increase the minimum contacted area between the ridges and the fusiform area during sliding. Thus, the bump could not be fully released, and making the force curve variation become smaller.

The measured voltage curves in real tests are shown in Figure 10b,d,f. Most phenomena discussed in the simulation cases could be verified here, like the peak number drops from 8 to 4 when the angle increases to 60°. The above results indicated that the grooved surfaces with different spatial period and inclined angle arrangement could be successfully discriminated by using the proposed flexible tactile sensor sliding motion and phase delay algorithm, thus may have potential in robotic grasping tasks for surface texture recognition.

## 5. Conclusions

This study develops a methodology using FEM modeling and the Phase Delay Algorithm to validate the flexible tactile sensor array for slippage and surface texture recognition in sliding motions. The structure and working principle of the tactile sensor array and its 3D FEM modeling are presented. The hyper-elastic Yeoh model is utilized to describe the material properties of PDMS, RTV adhesive, and conductive rubber utilized in the tactile sensors. For the sensor array sliding along the flat surface, both FEM simulation and experiments demonstrated that slippage occurrence and sliding direction can be determined based on the simulated normal force and measured voltages in the side resistors’ region. For surface texture recognition, the Phase Delay Algorithm and its calculation procedure are developed. Results also showed that the grooved surface with and without inclined arranged grooves can be successfully discriminated.

This study opens up the opportunity to study surface texture identification for a flexible tactile sensor array in real applications. Optimal structural design of the flexible tactile sensor array including electrode pattern’s design needs to be performed in future work. Further, the approach of using phase delay algorithm and artificial neural network for the developed tactile sensor array for robotic hand discrimination of unknown surface textures will also be conducted.

## Figures and Tables

**Table 1:** Material properties of rubber, RTV, and PDMS materials.

Material	Yeoh Model			Poisson Ratio
	*C*_1_	*C*_2_	*C*_3_	
Conductive rubber	0.5686	0.0540	−0.0181	0.47
RTV adhesive	0.5551	−0.0356	0.0027	0.48
PDMS	0.7997	0.2881	−0.0375	0.47 [27]

**Table 2:** Results of calculated spatial period and inclined angle in grooved surfaces.

	Spatial Period Discrimination (angle = 0°)				Inclined Angle Calculation (*D* = 1.2 mm)					
	Real D/mm	0.90	1.20	1.50	Real Angle	0°	15°	30°	45°	60°
Simulation	MAF | Error (Hz)	1.11 | 0.0%	0.78 | 6.0%	0.67 | 0.0%	*α* | Error	0.00° | 0.0%	11.51° | 23.3%	29.12° | 2.9%	44.10° | 2.0%	62.53° | −4.2%
	*D* | Error (mm)	0.90 | 0.0%	1.12 | 6.7%	1.50 | 0.0%	*D* | Error (mm)	1.14 | 5.0%	1.31 | −9.2%	1.16 | 3.3%	1.14 | 5.0%	1.23 | −2.5%
Experiment	MAF | Error (Hz)	1.06 | 4.5%	0.83 | 0.0%	0.67 | 0.0%	*α* | Error	0.48° | /	13.59° | 7.4%	29.90° | 0.3%	45.47° | 1.0%	61.50° | −2.5%
	*D* | Error (mm)	0.95 | −5.6%	1.20 | 0.0%	1.50 | 0.0%	*D* | Error (mm)	1.25 | −4.2%	1.22 | −1.7%	1.30 | −8.3%	1.31 | −9.2%	1.19 | 0.8%
